# Three Inferior Oblique Weakening Procedures for Management of Mild Hypertropia in Unilateral Superior Oblique Muscle Palsy

**DOI:** 10.18502/jovr.v19i4.14394

**Published:** 2024-12-31

**Authors:** Hajar Farvardin, Fatemeh Ebrahimi, Mohammadreza Talebnejad, Hadi Farvardin, Alireza Attar, Majid Farvardin

**Affiliations:** ^1^Poostchi Ophthalmology Research Center, Department of Ophthalmology, School of Medicine, Shiraz University of Medical Sciences, Shiraz, Iran; ^2^Student Research Committee, School of Medicine, Shiraz University of Medical Sciences, Shiraz, Iran; ^4^Hajar Farvardin: https://orcid.org/0009-0004-5196-3621; ^5^Majid Farvardin: https://orcid.org/0000-0001-9047-0329

**Keywords:** Fourth Cranial Nerve Palsy, Hypertropia, Incomitant Strabismus, Superior Oblique Palsy

## Abstract

**Purpose:**

To investigate the surgical outcomes of three different types of inferior oblique muscle weakening procedures in patients with mild hypertropia due to unilateral superior oblique muscle palsy.

**Methods:**

We reviewed surgical data of patients aged > 30 years with unilateral superior oblique palsy. The patients were categorized into three groups in terms of the surgical procedure: inferior oblique myectomy (IOM), inferior oblique recession (IOR), and inferior oblique disinsertion (IOD). Patients with preoperative hypertropia of 6 to 10 prism diopters (PD) were selected as mild cases for further comparison. Demographic data, preoperative and postoperative deviations in the primary position, contralateral gaze, contralateral elevation gaze, and ipsilateral tilt were considered for analysis. Complete success was defined as achieving postoperative orthotropia in addition to the resolution of diplopia and head tilt.

**Results:**

A total of 82 patients with an average age of 11.8 years were included in this study. The male-to-female ratio was 1.27, and the most common cause of palsy was congenital (89%). Fifty-six patients were treated with IOM, thirteen with IOR, and thirteen with IOD. The mean hypertropia correction was significantly better in the IOM group (9.1 PD) than in the IOR (7.1 PD) and IOD (7.5 PD) groups. Complete success was achieved in 80.3% of the IOM group, 69.2% of the IOR group, and 84.6% of the IOD group. The reoperation rate was 1.7% in the IOM group and 7.6% in the IOR group.

**Conclusion:**

Compared to IOR and IOD procedures, IOM was more effective in correcting hypertropia in patients with inferior oblique muscle overaction and mild hypertropia secondary to unilateral superior oblique palsy.

##  INTRODUCTION 

Superior oblique (SO) muscle palsy is the most common type of cyclo-vertical misalignment and cranial nerve palsy in strabismus clinics.^[1,2]^ Patients with SO muscle palsy typically present with hypertropia (HT) of the affected eye, which worsens in contralateral gaze and ipsilateral head tilt.^[3]^ Other clinical manifestations include compensatory abnormal head tilt, diplopia, and asthenopia.^[3,4]^

Despite the well-established understanding of SO muscle palsy, its treatment is still challenging due to the complexity of this condition. Surgical interventions are the mainstay of treatments for improving vertical and torsional deviations, diplopia, and torticollis.^[5,6]^ The choice of surgical technique depends on several factors, including the degree of torsion, the extent of HT in the primary gaze, and the degree of horizontal misalignment.^[7,8,9,10]^

Substantial research has reported the efficacy of inferior oblique (IO) muscle weakening procedures for patients with isolated SO muscle palsy and HT up to 15 prism diopters (PD) in the primary position.^[9,10]^ There are various surgical options available for IO muscle weakening, including anterior transposition, recession, myectomy, and disinsertion.^[3]^ The severity of HT, potential risks and benefits associated with each procedure, as well as the surgeon's experience and preference are important factors that must be considered when selecting the most appropriate option.^[7,8,9,10]^ Previous studies have evaluated the effectiveness and safety of different IO muscle weakening procedures for correcting HT, but none has specifically focused on patients with mild hyperdeviation. This study investigates the surgical outcomes of different IO muscle weakening procedures in patients with mild HT (6 to 10 PD) resulting from SO muscle palsy. Besides, the efficacy, complications, and success rates of these procedures will be compared.

##  METHODS

This study was designed as a retrospective review of patients with unilateral SO muscle palsy who were operated on from 1993 to 2022 by a single surgeon. Those with primary position HT of 6 to 10 PD were selected for further evaluation. Patients with any intraoperative evidence of superior rectus muscle contracture or SO muscle laxity were excluded from this study. Likewise, patients with bilateral SO muscle palsy, history of simultaneous surgery on another vertical muscle, previous surgery for vertical deviation, insufficient clinical records, and failure to attend the follow-up visits were also excluded. Patients with a minimum follow-up of six months were included in the study. Complete success was defined as attaining postoperative orthotropia and resolution of both diplopia and head tilt. Partial success was defined as postoperative vertical deviation of < 5 PD or decreased (but not resolved) head tilt. Surgical failure, on the other hand, was defined as postoperative vertical deviation ≥ 5 PD as well as persistent head tilt or diplopia.

Data collected for each patient included sex, age at the time of surgery, involved eye (paretic eye), etiology, presence or absence of diplopia, preoperative deviation in primary position, contralateral gaze, ipsilateral tilt, contralateral elevation, type and date of strabismus surgery, and follow-up data. Congenital etiology was considered in patients with a history of vertical strabismus or abnormal head posture since early childhood, if there were no other contributing factors. Traumatic etiology was considered if there was a positive history of head trauma due to a fall, traffic accident, or assault. Patients with SO muscle palsy for whom no identifiable etiological factor was found after thorough investigations were labeled as having an unknown etiology. All patients underwent a comprehensive ophthalmic examination, including ocular deviation measurement by prism cover test while maintaining fixation in the primary position at 33 cm with appropriate accommodative targets. Additionally, we evaluated ocular deviation in all nine gazes and head tilts.

**Table 1:** Demographic characteristics of patients with mild hypertropia due to unilateral superior oblique muscle palsy.

**Variable**	**IOM (** ***n*** ** = 56)**	**IOR (** ***n*** ** = 13)**	**IOD (** ***n*** ** = 13)**	***P*** **-value**
Gender: male/female (*n*)	35.21	4.9	7.6	0.11 ${\;}^{*}$
Paretic eye: right/left (*n*)	30/26	6/7	8/5	0.75 ${\;}^{*}$
Age at the time of surgery (year), mean ± SD	11.8 ± 11.2	10.6 ± 6.3	12.9 ± 11.9	0.75 ${\;}^{§}$
Duration of follow-up (month), mean ± SD	16.1 + 18.2	11.5 ± 14.8	19.5 ± 17.2	0.56 ${\;}^{§}$
Amblyopia, *n* (%)	5 (8.9%)	0 (0.0%)	1 (7.7%)	0.82 ${\;}^{†}$
Simultaneous horizontal deviation, *n* (%)	28 (50%)	7 (53.8%)	5 (38.5%)	0.69 ${\;}^{*}$
Etiology, *n* (%)				0.089 ${\;}^{†}$
Congenital Trauma Unknown	51 (91.1%) 2 (3.6%) 3 (5.3%)	9 (69.2%) 3 (23.1%) 1 (7.7%)	13 (100%) 0 (0.0%) 0 (0.0%)	
IOM, inferior oblique muscle myectomy; IOR, inferior oblique muscle recession; IOD, inferior oblique muscle disinsertion; n, number; SD, standard deviation ${\;}^{*}$ *P*-value by Pearson chi-square; ${\;}^{§}$ *P*-value by Kruskal–Wallis test; ${\;}^{†}$ *P*-value by Fisher exact test				

**Table 2:** Details of vertical deviation, diplopia, and head tilt before and after three different IO weakening procedures.

**Variable**	**Preoperative**				**Postoperative**				**Correction degree**			
	**IOM (***n*** ** = 56)****	**IOR (***n*** ** = 13)****	**IOD (***n*** ** = 13)****	***P*** **-value**	**IOM (***n*** ** = 56)****	**IOR (***n*** ** = 13)****	**IOD (***n*** ** = 13)****	***P*** **-value**	**IOM (***n*** ** = 56)****	**IOR (***n*** ** = 13)****	**IOD (***n*** ** = 13)****	***P*** **-value**
Primary position HT	9.3 ± 1.0 (6–10)	8.2 ± 1.9 (6–10)	7.7 ± 1.0 (6–10)	< 0.001 ${\;}^{MD§}$	0.2 ± 0.7 (0–5)	1.1 ± 1.8 (0–5)	0.2 ± 0.5 (0–2)	0.017 ${\;}^{\text{MR}§}$	9.1 ± 1.2 (5–10)	7.1 ± 2.1 (3–10)	7.5 ± 1.1 (6–10)	< 0.001 ${\;}^{\text{MD,MR}§}$
Contralateral gaze HT	14.7 ± 3.3 (10–30)	13.5 ± 3.8 (8–20)	11.7 ± 1.9 (8–15)	0.004 ${\;}^{\text{MD}§}$	1.0 ± 1.8 (0–8)	1.8 ± 2.5 (0–6)	0.5 ± 1.2 (0–4)	0.259 ${\;}^{§}$	13.7 ± 2.9 (7–25)	11.7 ± 3.4 (8–18)	11.2 ± 2.2 (8–15)	0.005 ${\;}^{\text{MD}§}$
Contralateral elevation HT	18.8 ± 3.8 (10–35)	19.2 ± 5.2 (10–30)	15.3 ± 2.3 (12–20)	0.003 ${\;}^{\text{MD,DR}§}$	0.4 ± 2.3 (0–15)	2.9 ± 4.1 (0–10)	1.1 ± 1.7 (0–4)	0.003 ${\;}^{\text{MR}§}$	18.4 ± 4.2 (10–25)	16.3 ± 5.7 (10–25)	14.2 ± 3.0 (11–20)	0.005 ${\;}^{\text{MD}§}$
Ipsilateral tilt HT	15.4 ± 3.6 (10–30)	15.7 ± 4.8 (10–25)	12.8 ± 2.0 (10–15)	0.026 ${\;}^{\text{MD}§}$	1.5 ± 2.7 (0–10)	2.2 ± 2.9 (0–6)	1.4 ± 1.8 (0–4)	0.549 ${\;}^{§}$	13.9 ± 4.3 (10–30)	13.5 ± 4.7 (9–20)	11.4 ± 2.1 (8–15)	0.055 ${\;}^{§}$
Diplopia, *n* (%)	6 (11%)	3 (23%)	4 (31%)	0.12 ${\;}^{†}$	1 (2%)	0 (0%)	0 (0%)	1 ${\;}^{†}$				
Head tilt, *n* (%)	46 -82%	10 -77%	10 -77%	0.75 ${\;}^{†}$								
Persisted						1(2%)	0 (0%)	0 (0%)				
Decreased						11(20%)	3(23%)	2(15%)				
Resolved						34(60%)	7(54%)	8(61%)				
IOM, inferior oblique muscle myectomy; IOR, inferior oblique muscle recession; IOD, inferior oblique muscle disinsertion; HT, hypertropia; PD, prism diopter ${\;}^{§}$ *P*-value by Kruskal–Wallis test; ${\;}^{†}$ *P*-value by Fisher exact test Further statistical analysis in *P * < 0.05 (statistically significant) by Bonferroni post hoc test ${\;}^{\text{MD}}$ Statistically significant comparison between IOM and IOD groups; ${\;}^{\text{DR}}$ Statistically significant comparison between IOD and IOR groups; ${\;}^{\text{MR}}$ Statistically significant comparison between IOM and IOR groups Hypertropia presented as mean ± SD (range) Diplopia, head tilt, and second operation presented in number and percentage (within each group)												

**Table 3:** Success rate and reoperation rate of three different IO weakening procedures.

	**IOM (** ***n*** ** = 56)**	**IOR (** ***n*** ** = 13)**	**IOD (** ***n*** ** = 13)**
Complete success: *n* (%)	45 (80.4%)	9 (69.2%)	11 (84.6%)
Partial success: *n* (%)	10 (17.8%)	3 (23.1%)	2 (15.4%)
Surgical failure: *n* (%)	1 (1.7%)	1 (7.6%)	0 (0.0%)
Reoperation: *n* (%)	1 (1.7%)	1 (7.6%)	0 (0.0%)
Hypertropia correction (%)	97%	86%	97%
IOM, inferior oblique muscle myectomy; IOR, inferior oblique muscle recession; IOD, inferior oblique muscle disinsertion; *n*, number			

**Table 4:** Previous comparative studies on patients with superior oblique muscle palsy treated with inferior oblique muscle myectomy, recession, and disinsertion.

**Comparative study**		**Preoperative HT (PD)**	**Postoperative HT (PD)**	**Correction in HT (PD)**	**Percentage of correction**
Bahl et al^[10]^	IOM (*n* = 43)	13.3	0.4	12.9	97%
	IOR (*n* = 42)	12.1	2.7	9.4	77%
Shipman et al^[11]^	IOM (*n* = 12)	15	1	14	93%
	IOR (*n* = 11)	10	2	8	80%
Akbari et al^[13]^	IOM (*n* = 34)	15.8	1.5	14.3	91%
	IOD (*n* = 28)	14.5	4.5	10	69%
Alajbegovic-Halimic et al^[14]^	IOR (*n* = 23)	8.7	1.3	7.4	85%
	IOD (*n* = 10)	5.9	1.5	4.4	75%
Current study	IOM (*n* = 56)	9.3	0.2	9.1	97%
	IOR (*n* = 13)	8.2	1.1	7.1	86%
	IOD (*n* = 13)	7.7	0.2	7.5	97%
IOM, inferior oblique muscle myectomy; IOR, inferior oblique muscle recession; IOD, inferior oblique muscle disinsertion; *n*, number; HT, hypertropia; PD, prism diopter.					

**Figure 1:**
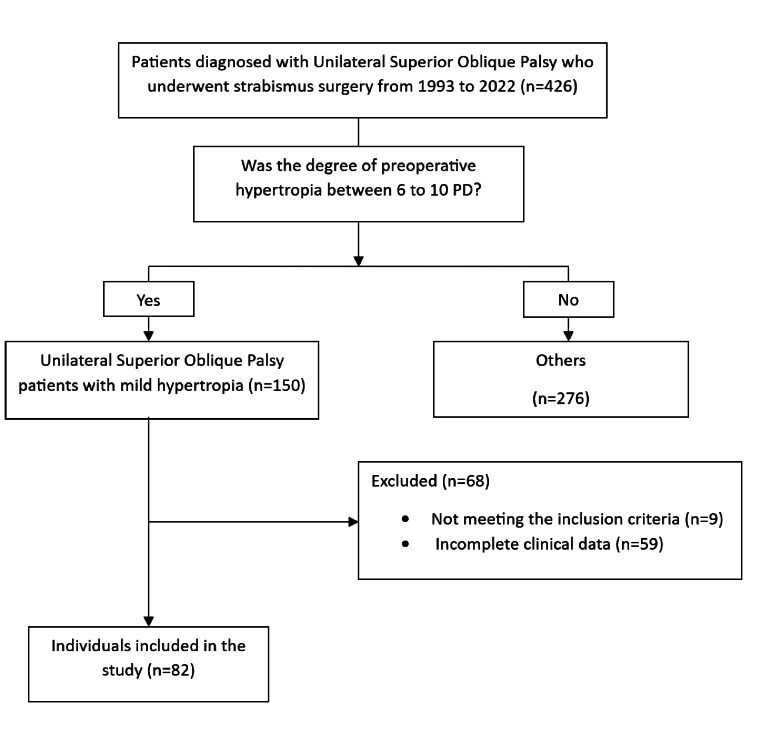
Participant flow chart.

In this study, all procedures were performed by a single surgeon (MF) under general anesthesia. Three different techniques were performed to manage SO muscle palsy: inferior oblique disinsertion (IOD), inferior oblique recession (IOR), and inferior oblique myectomy (IOM). In the first few years of the surgeon's experience, patients were randomly selected for IOR, IOD, or IOM. As time passed, considering the postoperative results, the surgeon preferred IOM over IOR, and IOD was reserved for cases with minimal HT ( < 8 PD in primary position).

In all procedures, a 6.0 silk traction suture (Ethicon, Johnson&Johnson) was used to expose the inferotemporal quadrant of the perilimbal conjunctiva, and the globe was kept in adduction and elevation. After a fornix inferotemporal peritomy was performed 10 mm from the limbus and the tenon's capsule and intermuscular septum were dissected, the IO muscle was exposed using a tenotomy hook under direct visualization. In the IOD technique, the muscle was simply cut flush to its insertion, and the proximal segment was slightly cauterized. Then, the muscle was allowed to retract spontaneously. In the IOR technique, the IO muscle was clamped adjacent to its insertion, then disinserted from the globe between the clamp and its insertion. After that, a 6.0 Vicryl suture (Ethicon, Johnson&Johnson) was passed through the IO muscle adjacent to the artery clamp with lock-bites at either pole. The two ends of the suture were then passed through the superficial scleral lamella 3 mm apart. Next, the anterior suture was inserted 4 mm posterior and 2 mm lateral to the temporal pole of the inferior rectus (IR) muscle (standard 10 mm IO muscle recession). In the IOM procedure, after the IO muscle was exposed, a hemostat clamp was applied approximately 8 mm from the insertion and held in place for a few seconds. Then, the muscle was separated from the scleral incision, and the segment between the clamp and the insertion was excised. Thermal cauterization was applied, and the muscle was allowed to retract freely.

This study followed all relevant tenets of the Declaration of Helsinki and its protocol was approved by the Ethics Committee at Shiraz University of Medical Sciences, Shiraz, Iran (ID: IR.SUMS.MED.REC.1401089).

### Statistical Analysis

All data were analyzed using the SPSS software (version 16.0, IBM). P < 0.05 was considered statistically significant for all tests. Categorical variables were summarized as numbers (percentages) and analyzed using Fisher exact test and chi-square tests. Numerical variables were presented as mean ± standard deviation (SD) and tested for normality using Kolmogorov–Smirnov test (P > 0.05 was considered as normal distribution). Parametric and non-parametric variables were analyzed by one-way analysis of variance (ANOVA) and the Kruskal–Wallis test, respectively. For significant results, post hoc multiple comparisons were performed using Bonferroni correction. Lastly, Spearman rank correlation coefficient was used to assess non-parametric bivariate correlation.

##  RESULTS

Out of the 82 eligible patients diagnosed with unilateral mild SO muscle palsy over a period of 30 years, 46 (56%) were male [Figure 1; Table 1]. The mean age of the patients at the time of surgery was 11.8 ± 10.6 years (range, 1–51 years). The most common cause of palsy in all participants was congenital (73 cases, 89%), followed by trauma (5 cases, 6%) and unknown etiology (4 cases, 5%). Patients were categorized into three groups based on their IO muscle weakening surgical procedure: 56 patients (68.3%) were treated by IO myectomy (IOM group), 13 patients (15.9%) were treated by IO recession (IOR), and 13 patients (15.9%) were treated by IO disinsertion (IOD). The mean follow-up period for all participants was 15.9 ± 17.5 (6–60) months. In Table 1, the demographic characteristics of the patients are stratified by the type of surgery.

**Before surgery, diplopia was detected in 13 patients (15.8%), and abnormal head tilt was noticed in 66 patients (80.5%). There were no significant differences between the three different surgical groups in terms of preoperative diplopia and abnormal head tilt (P = 0.12 and P = 0.75, respectively). After the operation, head tilt was completely resolved in 49 patients, decreased in 16 patients, and persisted in 1 patient.**

Table 2 presents the amount of HT in different eye positions before and after surgery across all study groups. Before surgery, the IOM group exhibited a higher HT in the primary position (9.3 PD) than the other two groups (8.2 PD in the IOR group and 7.7 PD in the IOD group). This difference was only significant between the IOM group and the IOD group (*P* < 0.001). After surgical correction, the mean residual primary position HT was greater in the IOR group (1.1 PD) than in the other two groups (0.2 PD in the IOM group and 0.2 PD in the IOD group). This difference was only significant between the IOR group and the IOM group (*P* < 0.001). The IOM group demonstrated the greatest HT correction in the primary position (9.1 PD), which was significantly greater than that in the IOD group (7.5 PD, *P *= 0.01) and the IOR group (7.1 PD, *P *= 0.01). In both contralateral gaze and contralateral elevation, the IOM group achieved the highest correction, which was statistically significantly greater than that of the IOD group (*P *= 0.01 and 0.005, respectively). The IOM group also exhibited a greater HT correction in ipsilateral tilt than did the other two groups; however, there was no significant difference among the study groups in terms of the resulting postoperative correction.

For each patient, the percentage of correction was calculated by the extent of HT correction divided by the extent of preoperative HT. The percentage of correction for the IOM group, IOR group, and IOD group was 97%, 86%, and 97%, respectively. In the IOD group, surgery led to complete success in 84.6% and partial success in 15.4% of patients. In the IOR group, the rates of complete success, partial success, and surgical failure were 69.2%, 23.1%, and 7.6%, respectively. In the IOM group, the rates of complete and partial success were 80.4% and 17.8% respectively; and 1.7% of operations led to surgical failure. Overall, primary strabismus surgery was successful in 80 patients and only 2 patients (2.4%) required a second operation for their residual hyperdeviation. Both of these individuals were treated by contralateral IR recession with and without posterior fixation sutures in order to address five PD residual HT. The reoperation rate was 1.7% in the IOM group and 7.6% in the IOR group. Two other patients underwent a second strabismus surgery for their residual horizontal deviation [Table 3].

We also investigated the relationship between the amount of HT correction followed by IO weakening surgery and preoperative HT in our study groups. Spearman rank correlation coefficient showed that improvement in HT was proportional to the extent of preoperative HT before performing the IO weakening procedure: the larger the preoperative hyperdeviation, the greater the effect of surgery. There was a positive correlation between preoperative vertical deviation and HT correction after surgery (*P* < 0.001, correlation coefficient = 0.903).

##  DISCUSSION

Based on our results, over one-third of the patients who were operated on due to unilateral SO palsy fall within the mild group, in which the preoperative HT in the central gaze is 6 to 10 PD. Choosing an appropriate IO muscle weakening procedure to treat these patients is of great value.^[3,4]^ Over time, different surgical options have been introduced for weakening the IO muscle. Examples include IO myectomy, IO disinsertion (myotomy), IO recession, IO anterior transposition, and combined resection and anteriorization of the IO muscle.^[9,10][11][12][13][14][15,16,17,18,19][20]^ These techniques have achieved various amounts of HT correction in different studies. Transposition of the IO muscle with or without resection can correct larger amounts of hyperdeviation and is, therefore, usually considered for more severe cases, but all other mentioned procedures have been utilized to correct mild HT in unilateral SO muscle palsy.^[15,16,17,18,19]^ In this study, 82 patients with mild HT due to unilateral SO muscle palsy were treated by three different IO muscle weakening procedures: IOM, IOR, and IOD.

Although all enrolled patients in this study had a preoperative primary position HT ≤ 10 PD, the HT was significantly greater among patients treated by IO myectomy. This finding reflects the surgeon's preference for IO myectomy in patients with larger preoperative hyperdeviation. In the current study, IO myectomy led to slightly better postoperative results compared to IO recession. Thus, HT correction in the primary position was significantly better in the IOM group than in the IOR group. The mean postoperative HT was 0.2 PD in the IOM group, which was significantly lower than that of the IOR group (1.2 PD). The reoperation rate was 1.7% in the IOM group and 7.6% in the IOR group. Moreover, the mean HT correction in the IOM group was significantly better than the IOD group in the primary gaze, contralateral gaze, and contralateral elevation gaze.

All previous studies have indicated that IO myectomy is slightly more effective than IO recession as far as HT improvement in the primary gaze is concerned.^[9,10][11]^ In their study on 85 patients with unilateral SO palsy, Bahl et al showed that the mean HT correction after IOM and IOR was 12.6 PD and 9.2 PD, respectively.^[10]^ The mean postoperative HT of patients who were treated with IOR was 2.74 PD in the primary position, and six patients (14%) needed a second surgery due to residual vertical deviation. They further noted that differences between IOM and IOR were even more pronounced in patients with preoperative deviation up to 15 PD. The authors concluded that IO myectomy would be a better and simpler surgical option in patients with unilateral SO muscle palsy.^[10]^ Similarly, Shipman et al noted that IO myectomy was more effective than IO recession in correcting HT in patients with SO muscle palsy (14 PD correction in the IOM group vs 8 PD correction in the IOR group).^[11]^

IO disinsertion is thought to be a weaker version of the IO myectomy technique.^[12,13,14,15]^ Yanyali et al showed that IO disinsertion led to unsatisfactory results in patients with larger preoperative HT.^[12]^ In their study, the mean preoperative and postoperative HT in patients who were treated by IOD was 22 PD and 9 PD, respectively, and the reoperation rate was 27.2%.^[12]^ Akbari et al compared IO myectomy and IO disinsertion in patients with unilateral SO palsy.^[13]^ In their subgroup analysis among patients with HT up to 15 PD, IOM and IOD reduced HT by 8.8 PD and 7.6 PD, respectively. Although the mean HT correction and the mean postoperative HT were both better in the IOM group, the results were not statistically significant in this subgroup of patients.^[13]^ However, in our study on patients with mild HT caused by unilateral SO muscle palsy, the HT reduction achieved in the IOM group was significantly greater than that in the IOD group in all studied gaze positions (primary position, contralateral gaze, and contralateral elevation).

Although small-angle HT can be treated using prisms, IO muscle weakening surgery offers the advantage of reducing lateral incomitance, and the reported rate of overcorrection following this procedure is slightly low.^[4,21]^ Hendler et al studied 25 patients with preoperative HT up to 10 PD who were all treated by IO recession.^[21]^ The mean HT reduction was 5.4 PD and 4% of patients experienced overcorrection. In our study on 82 patients with mild HT who were treated with three different IO weakening procedures, 8 patients experienced residual HT up to 5 PD but none showed overcorrection in their last follow-up. Recently, a new study reported successful results with IO muscle belly transposition in 10 patients with IO muscle overaction and small HT of < 5 PD.^[20]^

Some studies have noted the self-grading nature of IO weakening procedures.^[7,9,11]^ Shipman et al stated that both IO myectomy and IO recession are largely self-grading; therefore, the higher the preoperative HT, the greater the surgical outcome.^[11]^ Another study similarly confirmed the relationship between preoperative vertical deviation and the effect of IO myectomy.^[9]^ Our results further indicated that there is a positive correlation between the extent of preoperative HT and the degree of HT correction following IO muscle weakening surgery.

The current study has several limitations mostly stemming from its retrospective nature. We could not overcome the problem of uneven distribution of patients among three different groups of IO weakening. Most of the enrolled patients were treated by IO myectomy due to the surgeon's preference. This could have been a possible source of bias in this study since the surgeon stopped performing IO recession after about 15 years due to its poor postoperative outcomes. Although the three groups undergoing IO weakening procedures were not significantly different in terms of age, sex, and etiology, the mean preoperative deviation was greater in the myectomy group. Considering the self-grading nature of IO weakening procedures, it was challenging to compare the resulting HT corrections achieved by each method. Furthermore, it is difficult to make an accurate comparison between the results of IO recession due to subtle differences in the details of the reported surgical techniques. Another limitation was the diverse etiologies for SO palsy in our participants. To better investigate the differences among various IO weakening procedures, it may be rewarding to conduct parallel-group randomized controlled trials.

Various IO muscle weakening procedures are available for correcting HT in unilateral SO muscle palsy. In the present study, IO myectomy was more effective in correcting HT compared to IO recession and IO disinsertion for patients with IO overaction and mild HT secondary to unilateral SO palsy. To the best of our knowledge, the current study is the first to compare three different IO weakening procedures in the management of mild HT (6 to 10 PD) in cases with unilateral SO muscle palsy. Based on our retrospective study, IO myectomy showed slightly better outcomes compared with IO recession. Regarding IO disinsertion, it is suggested to reserve this technique as an appropriate surgical option in cases with lower preoperative HT ( < 8 PD).

##  Financial Support and Sponsorship

This work was supported by the Shiraz University of Medical Sciences (IR.SUMS.REC.1401089).

##  Conflicts of Interest

None.
